# Study protocol for Goodform - a classroom-based intervention to enhance body image and prevent doping and supplement use in adolescent boys

**DOI:** 10.1186/s12889-020-8166-2

**Published:** 2020-01-14

**Authors:** Joanna Rachel Doley, Siân Alexandra McLean, Scott Griffiths, Zali Yager

**Affiliations:** 1 First Year College, Victoria University, Footscray Park, Footscray, Australia; 2 College of Arts and Education, Victoria University, Footscray Park, Footscray, Australia; 3 School of Psychological Sciences, University of Melbourne, Parkville, Australia

**Keywords:** Body image, Boys, Steroids, Supplements, Prevention, Intervention, Study protocol

## Abstract

**Background:**

Very few programs aimed at improving body image among adolescent boys have been effective, and there is still no clear evidence as to what will work for universal prevention of eating disorders and body dissatisfaction with this group. We combined two previously efficacious programs and used a design thinking framework to optimise program content alongside potential end-users including adolescent boys, teachers, parents, and experts. Goodform is a four-session universal program that aims to reduce body dissatisfaction and prevent the use of muscle-building supplements among 14-to-16 year old adolescent boys.

**Methods/design:**

Goodform will be trialled using a cluster randomised controlled trial (RCT) conducted in Australian schools, with Year 9 boys as participants. The intervention is teacher-delivered. Data will be collected at three time points: baseline, post-intervention, and follow-up (2 months). Three primary outcome constructs will be examined, including body dissatisfaction (Male Body Attitudes Scale-Revised) and attitudes towards appearance and performance enhancing substances (APES; Outcome Expectations for Steroid and Supplement Use, Intentions to use APES) and actual use of APES at each time point. Three secondary outcome constructs will be examined, which are social norms for APES (adapted Peer Norms Scale), negative body talk (Male Body Talk Scale), and internalisation of and pressure to attain appearance ideals (Sociocultural Attitudes Towards Appearance Questionnaire-4 Revised). Internalisation of appearance ideals will also be examined as a mediator of change in primary outcomes. Teachers will provide data on adherence to lessons, student engagement/enjoyment, and understanding of the content.

**Discussion:**

The GoodForm RCT will trial a novel, generalizable, and extensively developed program intended to improve boys’ body image and reduce actual and intended APES use. We anticipate that it will provide a novel contribution to the field of boys’ body dissatisfaction prevention.

**Trial registration:**

This trial was retrospectively registered with the Australian and New Zealand Clinical Trials Registry on May 14th 2019, registration number ACTRN12619000725167.

## Background

Goodform is a four-lesson classroom-based educational program for boys aged 14–16 aimed at improving body image, reducing positive outcome expectations for steroid use, and reducing intentions to use appearance and performance enhancing substances (APES). The program is based on two highly successful existing programs; ATLAS [1] and The Body Project [2], and uses both a dissonance-based approach and a social norms approach to achieve outcomes.

The intervention will be delivered by Health and Physical Education (HPE) teachers of Year 9 boys in secondary school, during weekly face-to-face HPE classes at participants’ schools. All teachers will receive a facilitator workbook (specifically designed for this program) with structured activities and explanations for how to deliver the program. Resources to support delivery of activities are also provided. Attendance of participants will be monitored using attendance sheets for each lesson. Adherence to session content will be monitored using a self-report checklist completed by the teacher following each lesson.

Participants will be allocated to the intervention or control group through cluster randomisation, at the school level. Intervention participants will complete questionnaires at baseline, receive the four-lesson program, delivered once-per week, and then complete 1 week post-intervention, and follow-up (2 months after post-test) self-report questionnaires. Participants in the control condition will complete questionnaires at parallel timeframes, but will not receive the intervention. Comparison of change over time between the intervention and control groups will inform evaluation of program efficacy. We hypothesise that, relative to the control group, the intervention group will show improved scores on primary outcomes (body image, attitudes towards APES, intentions to use APES, use of supplements) and secondary outcomes (body talk, social norms for APES, body talk, internalisation of appearance ideals).

### Literature review

Body dissatisfaction and related psychological disorders, including eating disorders, are now widely recognised as issues for boys and men [3]. While prevention of body dissatisfaction and eating disorders among women and girls has made a great deal of progress since research on this topic first began in the 1980’s, little evidence exists regarding appropriate approaches and effective programs for boys and men. Several high-quality reviews and meta-analyses of body dissatisfaction and eating disorder programs exist [4–7]; however, within this literature, very few prevention programs have been designed specifically for boys.

The theoretical basis for prevention programs for males varies. Dissonance-based approaches are popular and have produced notable improvements in body dissatisfaction [8–10] and eating disorder symptoms [8, 9], with small-to-large effect sizes (*d* = 0.30–1.00) among adult men. Programs that have improved both eating disorder symptoms and body image in boys include a media literacy program [11], and a mixed-approach program including developmental, psychoeducational, body acceptance, and media literacy content [12]. However, findings appear less consistent in boys than in girls. For example, while an early trial of Media Smart [11] demonstrated improvements in body image and eating disorder symptoms, a later trial of the program [13] demonstrated significant reductions in body dissatisfaction among boys from pre-post, but found no change for weight and shape concern or dietary restraint. Adding to the complexity of research in this area, it is also common for authors to use combined approaches, using two or more strategies such as media literacy and a focus on the influence of peers [14–16], making it unclear as to which specific approaches and/or combinations may produce the most favourable outcomes.

In order to address the lack of knowledge about the most promising approaches and programs for boys, it may be necessary, at least initially, to design single-gender programs specifically for boys. Although co-educational universal body image and eating disorder programs are more common (e.g. [10, 14, 17, 18]) than programs specifically for boys or men, some programs have been designed for, and delivered to, male-only audiences [8, 9, 19–21], with some success, particularly among adult men. In previous body image research in a co-educational learning environment, either the boys or the girls improve; improvements are rarely observed for both genders [7]. A focus just on boys is therefore suggested in order to determine exactly what works for males, even though in the longer term, co-educational programs are more practical for the majority of school and university environments, and are important in achieving broad societal change, as opposite-sex peers play an important role in reinforcing and perpetuating sociocultural appearance ideals and pressures more broadly [22, 23]. In addition to these considerations, our other work has found that those who have higher levels of body dissatisfaction are more likely to indicate that they would prefer a single-sex environment for body image intervention programs [7]. Similarly, in the clinical space, some researchers suggest that male-only groups will allow boys and men to voice their concerns and be more vulnerable than in environments where women are present, and that the impact of realising that body concerns are normal among men is valuable in improving outcomes for men [24].

In addition to delivery environment, it is also important to examine the impact of the facilitator of intervention programs. Co-educational, and male-focussed programs have typically been delivered by a researcher [11, 12, 14, 15, 21, 25, 26], with the exception of The Body Project, which is typically peer-delivered [8–10]. At present, it appears that *who* delivers the program may be unimportant; no consistently positive or negative outcomes in intervention efficacy according to whether the facilitator was a researcher or a peer, or the gender of the facilitator, are apparent. Teacher-delivered interventions are less common [17] which may be due to the practicalities of training teachers in the program activities, and body image and eating disorders if they are unfamiliar with current literature.

There is now a well-recognised need to incorporate the perspectives of end-users into the design of intervention programs in order to ensure that what is developed meets their needs. Several prevention programs describe developing materials using collaborations between stakeholders and researchers [17], focus groups [14] or submitting activities to the target population for feedback [8, 9, 19]. This may be particularly important when designing or adapting materials for males, as the literature on the nature and experience of body image and eating disorders in men and boys is limited compared with the same topic for women and girls. Design thinking [27] and participatory action research [28] are both structured approaches to developing research that incorporates the views of the intended audience or recipients. We incorporated both of these approaches in the design of the Goodform intervention.

Design thinking gives a structured process for designing a product (or intervention) that takes the needs, attitudes, and feedback of end-users (in our case, end-users are adolescent boys, their teachers, and parents) into consideration [27, 29]. Design thinking approaches are similar to Community-based Participatory approaches, and involve: 1) considering the needs of end-users, and 2) incorporating the feedback and needs of end-users in an iterative process of product or intervention development to ensure that the end product will be usable and effective.

### Preliminary studies

We conducted three studies in the course of developing our intervention. Two were conducted by Masters-level students and are published in student theses [30, 31], and one was conducted by the research team [32].

The first study examined parent attitudes towards, and knowledge of, APES and body image [30]. Participants were parents of boys aged 14–17 (*N* = 7) and took part in semi-structured interviews. Some of the main findings that informed the intervention content of Goodform were that parents noted the lack of (but potential benefit from) education about APES within schools, and that this education should include evidence-based information with high source credibility.

The second study was a pilot test of a digital tool and video intended for the first lesson of Goodform [31]. Participants were 52 boys, with 28 trialling an initial version and 24 trialling a post-feedback optimised version. Generally, the content was acceptable although feedback indicated basic concepts should be explained in more detail. The majority of issues were practical or technical in nature and resulted in improved on-task behaviour (e.g., asking serious questions about the task, and reductions in off-task behaviour such as fidgeting) and engagement following resolution of these issues. Specifically, workbooks for both students and teachers were produced to help guide users through the task, errors in the tool itself were fixed, and content was added to the digital tool to allow for more student exploration of the topic.

The third study was a mixed-methods study which broadly focused on the process of developing a body image prevention program for boys [32]. This study incorporated feedback and findings from the two studies described above, in addition to feedback from in-depth interviews with boys, and questionnaire feedback from body image experts, with the aim of identifying effective strategies for developing boys’ body image programs. Five themes were identified that would benefit end-users (boys, their parents, and teachers) of body image programs, such as an awareness of social norms for boys, understanding the importance of authority and credibility of information, increasing interactivity of projects, and having a good understanding of classroom practicalities when delivering interventions.

### Study objectives and research questions

The 4-session Goodform program aims to engage boys to critique supplement use and societal muscular ideals in order to reduce body dissatisfaction and prevent APES use among adolescent boys. The present study aims to examine the efficacy of Goodform relative to a waitlist control condition for achieving the primary outcomes of reducing body dissatisfaction, favourable attitudes towards appearance and performance enhancing substances, and actual use of APES in mid-adolescent boys. Change across time in secondary outcomes of social norms for APES, negative body talk, and internalisation of and pressure to attain appearance ideals will also be examined. Finally, the study aims to examine if change in internalisation of appearance ideals mediates change in primary outcomes.

## Methods

### Study design

We will use a cluster randomised-controlled trial to evaluate Goodform. Participants will be randomised at the school level to either the intervention or waitlist control condition. We will measure study outcomes at three time points: baseline, post-test, and follow-up (2 months post-test). Schools in the waitlist control condition will implement the program following completion of the follow-up questionnaire.

### Intervention design

GoodForm is a program for adolescent boys aged 14–16, that aims to improve body image, and reduce intended and actual use of APES. We decided to focus only on boys in order to further understand the practical and theoretical strategies that will enhance body image and prevent supplement use in a single-gender environment. The program is intended to be delivered by HPE teachers within the classroom setting. The theoretical focus on supplements and steroids was informed by prior work demonstrating relationships between body image, supplement use, and gender norms, whereby boys were found to be more likely to use APES when they were also more dissatisfied with their body [33, 34]. We theorise that improving body image will reduce intentions to use supplements, as boys will have increased satisfaction with their appearance and functionality of their body, that will in turn, reduce their desire to use supplements. We also theorise that programming to reduce supplement use will act as cognitive dissonance against the societal muscular ideal, which will in turn improve body image. This dual focus is expected to enhance the outcomes of the intervention program in relation to the primary goal, to reduce intentions and use of APES.

To build on the existing evidence base, we chose to combine and optimise existing programs. We reviewed the literature on boys’ body image programs and found two programs that demonstrated some efficacy with adolescent boys. The Athletes Training and Learning to Avoid Steroids [ATLAS] program was one of the first to report a positive impact on the body image of adolescent male athletes in the USA [35]. The fourth and second authors (ZY and SM) conducted a replication of the ATLAS program in an Australian boys’ school and found acceptable outcomes on body image measures [36]. The Body Project: More than Muscles [8] has also been highly effective in improving body image among University/College aged male groups. We adapted elements of both programs to suit a universal, non-athlete adolescent audience. The combined intervention therefore takes a cognitive dissonance approach to critique the hyper-muscular ideal and the use of supplements and steroids, adopted from The Body Project, and social learning theory [37] approach adopted from ATLAS. The team added a focus on social norms, based on the success of this theoretical framework in interventions for alcohol use and stigma [38].

Dissonance-based approaches to body dissatisfaction interventions are theorised to work by creating a divide between how an individual behaves and their internalised attitudes; thus creating cognitive discomfort for the person. The discomfort can be resolved by the individual changing their attitudes to align with the behaviour [39]. In the context of the hyper-muscular ideal, participants will be asked to behave in a way (e.g., by arguing critically, role playing, or producing media critiques) that criticises this ideal; as such, the divide between internalisation of this ideal and their behaviour will create discomfort for the person and result in attitude change. In addition to the dissonance-based approach, social learning theory forms a basis for the program. Social learning theory posits that learning is acquired through observing others’ behaviour; through this observation, the behaviour is processed by the observer and becomes influential in shaping future behaviour [37]. In the context of body image and hyper-muscularity, teachers and students will model behaviours through critiquing APES and the hyper-muscular ideal. A social norms approach will also form the basis for specific sections of the intervention. The social norms approach posits that attitudes and behaviours are influenced by what is perceived to be acceptable and appropriate behaviours among meaningful others (e.g., well-liked peers [38];). As such, we theorise that by creating a classroom culture that opposes APES use and the hyper-muscular ideal, group norms towards these behaviours and ideals will change and students will adjust their attitudes and behaviours accordingly.

In order to combine and optimise the programs, we used a design thinking approach. Initially, all authors of the current paper, experts in body image, reviewed the content of the existing programs, obtained from the original authors, and the published papers reporting on these programs. We then held a workshop where we debated the importance, and developmental appropriateness of each of the activities in the existing programs, until we reached consensus on the activities that should be included. The fourth author (ZY) then used her expertise in education to create a proposed draft of the combined program. We worked with a design agency who created the branding and persona of the Goodform program, based on design thinking. The agency interviewed a number of adolescent boys to obtain the boys’ perspective on effective health education programs and school content relating to body image. Responses were presented to the research team, who adopted the suggestions provided in developing the program, such as the use of humour, language, and using a direct approach. As described above, the research team also conducted a range of user interviews with boys, parents, and teachers, described elsewhere [32]. Outcomes from these interviews, considered alongside existing literature, were used to develop a range of guiding principles for boys’ body image programs, which helped to guide and shape the outline of the Goodform program, as shown in Table 1. We intended for GoodForm to be a teacher-delivered intervention to maximise dissemination and generalisability. The intention was for the more complex body image content, and difficult aspects of the program to be conveyed in interactive tools and short films, rather than relying on teacher education and training to enable them to deliver the program.

**Table 1:** Overview of the GoodForm Program

Session	Activities	Adapted from:
Session 1: Cultural Ideals	1) Introduction 2) Video: Pressure to conform to the cultural ideal 3) Digital Tool and worksheet: Define and critique the cultural ideal for men 4) Worksheet: How do we challenge this ideal? Homework: Write your advice to a younger boy	Drawn from The Body Project: More than Muscles
Session 2: Supplements and Steroids	1) Discussion: Read through Homework Task 2) Demonstration: Balloons and steroids 3) Jigsaw Inquiry: What are the side effects of supplement use? 4) Jigsaw Activity: Critique Supplement Ads 5) Video: What will people think?	Drawn from the ATLAS program and extended to include other muscle building supplements.
Session 3: Critiquing Supplement use	1) Quiz: Recap previous content 2) Jigsaw Activity: Role Plays to counter the use of supplements and steroids 3) Jigsaw Assignment: Develop a media campaign against steroid use	Drawn from the ATLAS program and extended to include other muscle building supplements.
Session 4: Advocacy and Activism	1) Presentation of media campaigns 2) Advocacy and Activism: Top 10 Worksheet 3) What next? Challenge Yourself	Drawn from the ATLAS program and combined with the concept of advocacy from The Yager Body Project.

The first lesson of the Goodform program was based on the initial discussion from the first lesson of The Body Project. We worked with the design agency to develop this as an interactive online tool and to align this activity with the guiding principles of 1) privacy and a safe space, and 2) interactive tools and multimedia [32]. Illustrations were commissioned to guide the narrative and facilitate boys’ progress with the tool on their own or in pairs, reporting their responses in their worksheet. We tested the tool with three successive classes of year 7 and 9 boys [31], and made several changes to the wording and format of the content and worksheets to enhance comprehension of the messages intended in the program. This interactive tool is available for viewing at www.goodform.org.au.

In collaboration with a professional media agency, we also developed a short film for Goodform to clearly convey the idea that muscle building supplement use is not normal, acceptable, or advisable. This messaging was intended to contribute to the social norms component of the program (i.e., that using muscle building substances is perceived as unacceptable by important others). The film conveys how friends, girlfriends, parents, and sports coaches might react to discovering that the adolescent boy has been using an unnamed muscle building substance. We left the exact substance open to interpretation so that boys would interpret the film based on their current knowledge and experiences. Public health communication and social marketing campaigns often employ emotional tactics to create behaviour change. We used the literature from these research areas to inform the development and scripting of the film.

### Study population and recruitment

**Fig. 1:**
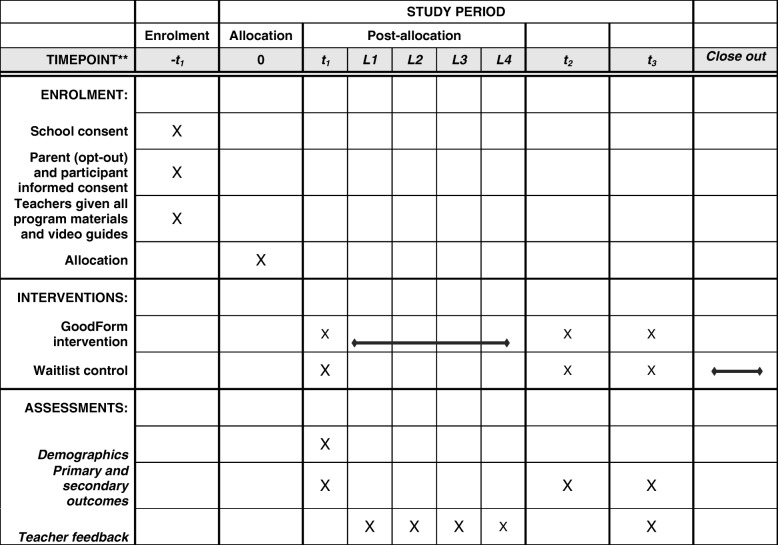
Schedule of enrolment, interventions, and assessments

Year 9 (age 14–16) boys from Australian schools will comprise the study population. Inclusion criteria is being a year 9 boy attending a school who has consented to participate in the trial. There are no exclusion criteria. Schools will be recruited by a member of the research team contacting principals and HPE teachers, to invite them to agree to participate in the study and deliver the Goodform lessons as part of their HPE curriculum. To minimise administrative load for schools, we will use informed opt-out consent for parents and adolescent boys. We will use a thorough process to ensure that participants and their parents have sufficient opportunity to understand the research and ask questions of the research team. Three weeks prior to data collection, information sheets and explanatory videos about the project will be given to parents of all participants, and they will be encouraged to contact the researchers if they have any questions about their involvement in the project. To opt their child out of the research, parents will fill in an electronic opt-out consent form (included with the information sent to them). Boys will also be given an information sheet and an explanatory video aimed specifically at them (i.e. with age appropriate language), and encouraged to ask questions of the researchers or discuss the program with their parents. Boys can choose not to complete the questionnaire if they do not wish to participate. Complete details of the study procedure, including enrolment, intervention, and recruitment are displayed in Fig. 1.

Health and physical education teachers who deliver GoodForm will also provide feedback data for the project, should they consent to do so. HPE teachers will be recruited through direct contact or approached by their school principal, and will indicate their consent to run the program and complete lesson feedback using an electronic consent form, which will be returned to the research team. Principals will provide consent for the school to participate using an electronic consent form, however they will not be asked to provide any data for the study.

### Allocation and blinding

Participants will be allocated at the school level by a member of the research team using simple randomisation on a computer program with a 1:1 ratio (intervention:control). Neither the participants nor the researchers will be blind to the intervention condition.

## Measures

All measures are self-report measures and were examined for suitability for adolescent males using the Simple Measure of Gobbledygook [40]. All measures were found to be suitable for a reading age of approximately 11 years and up. In total, boys’ questionnaires take approximately 15 min for each time point, with teacher questionnaires taking approximately 15 min in total.

### Body image

Two variables will assess the primary outcome construct of body image. These will be measured at all three time points. The Male Body Attitudes Scale [41, 42], a self-report measure that contains two subscales; muscularity dissatisfaction (7 items) and body fat dissatisfaction (5 items) will assess body image. Responses to items from both subscales are indicated on a 5-point Likert-type scale from 1 (*Never*) to 5 (*Always*), with higher scores indicating greater dissatisfaction. The mean score for each subscale will be used for analyses. Example items include “I think I have too little muscle on my body” (muscularity dissatisfaction subscale) and “I think my body should be leaner” (body fat dissatisfaction). Scores on the muscularity and body fat dissatisfaction subscales have demonstrated high internal consistency and construct reliability in young adult men [41], and have been validated in other body image research involving adult men [43, 44].

### Sociocultural appearance attitudes

The 28-item Sociocultural Attitudes Towards Appearance Questionnaire – 4 – Revised [45] will be used to assess appearance ideal internalisation and perceived pressure to achieve appearance ideals. Two subscales, internalisation thin/low body fat (two items, example item: “I think a lot about looking thin”) and internalisation muscular (four items, example item “It is important for me to look muscular”) will be used as secondary outcome measures and as mediators of change in muscularity and body fat dissatisfaction. The other subscales - pressures – media (five items, example item “I feel pressure from the media to look in better shape”), pressures – family (four items, example item “Family members encourage me to get in better shape”), pressures – friends (seven items, example item “My peers encourage me to increase the size or definition of my muscles”), internalisation – general attractiveness (two items that are reverse scored, example item “I don’t really think much about my appearance”), and pressures – general attractiveness (four items, example item “I feel pressure from my peers to improve my appearance”) will be used as secondary outcome measures. Responses to items for all subscales are indicated on a 5-point Likert-type scale from 1 (*Definitely Disagree*) to 5 (*Definitely Agree*) with higher scores indicating greater levels of internalisation or pressure. The mean score for each subscale will be used in analyses. Scores on all subscales of the SATAQ-4-R have good internal consistency in men [45] and have displayed good convergent validity (i.e., medium to large correlations) with other measures of body dissatisfaction and ED symptoms in men [46].

### Attitudes towards steroids

Outcome expectations for using steroids (OE-AAS; 4 items, [47]) and intentions to use steroids (I-AAS; 5 items, [47]) will be used to assess the primary outcome construct, attitudes towards APES, administered at all time points. Responses to both scales are indicated on a 7-point Likert-type scale, from 1 (*Strongly Agree*) to 7 (*Strongly Disagree*), with higher scores indicating less favourable attitudes towards steroids. The mean score for item responses from both subscales will be used in analyses. Scores for both scales have good to excellent internal consistency in men (α = .94 for OE-AAS and α = .92 for I-AAS) and evidence of good convergent validity, as demonstrated by correlations of .37–.44 with the Drive for Muscularity Scale [47].

### Use of supplements and steroids

Our final primary outcome is actual use of supplements and steroids, measured by two single items asking if participants have used either a) anabolic steroids within the past 3 months or b) supplements to build muscles or burn fat within the past 3 months. Responses to the items are indicated on a binary scale from 0 (*no*) to 1 (*yes*). Due to anticipated low baseline rates of use of supplements and steroids, statistical analyses are likely to be under-powered to detect reductions in use. However, frequency of use will be reported for this primary outcome variable.

### Social norms for using APES

Participants’ perceptions of social norms for using supplements and steroids (i.e., perceived prevalence, importance and acceptability of APES use among their peers) form three variables measuring the secondary outcome construct of social norms for APES. Items for these variables were adapted from Ling et al.’s [48] Peer Norms Scale of Physical Activity. Items concerning prevalence (example item: “How many of your close friends do you think take muscle building supplements?”) are measured on a 4-point scale, from 1 (*None*) to 4 (*All*), with higher scores indicating greater perceived prevalence of APES use. Items concerning importance (example item: “How important do you think it is to your close friends to avoid taking muscle building supplements?”) are measured on a 3-point scale, from 1 (*Not At All Important*) to 3 (*Very Important*), with higher scores indicating greater importance of avoiding APES. Items concerning acceptance (example item: “My friends would disapprove if they saw me using steroids.”) are measured on a 4-point scale, from 1 (*Disagree a lot*) to 4 (*Agree a lot*), with higher scores indicating less perceived acceptance of APES use. Scores on the Peer Norms Scale of Physical Activity, from which the scales in the present study were adapted have acceptable internal consistency α = .72, and acceptable one-week test-retest reliability of .78 [48].

### Negative body talk

Our final secondary outcome for boys will be negative body talk, the tendency to talk about concerns about fat and muscularity, assessed with the Male Body Talk Scale [49]. The scale comprises two subscales; muscle talk (6 items) and fat talk (10 items), and is measured on a scale from 1 (*Never*) to 7 (*Always*) with higher scores indicating more negative body talk. The subscales have excellent internal consistency in men (Muscle Talk α = .95, Fat Talk α = .92) and good evidence of convergent validity, with correlations between .20 and .58 on measures of body dissatisfaction and muscle dysmorphia [49].

### Teacher feedback

While not part of primary or secondary analyses, we will also examine teacher feedback scores for each topic within the program, using a scale adapted from McLean, Wertheim, Marques, and Paxton [50]. Four items for each topic measure level of student engagement, level of student understanding, the degree to which the activity was covered, and perceived activity success and items are rated from 1 (*not at all/low*), to 3 (*fully/high*). Optional qualitative feedback – specifically, any suggested changes for the lesson, and open-ended comments, may also be included.

### Data collection

All of the data for this study will be collected at the schools involved. Data collection will occur in the presence of the teacher facilitating the program. All teachers will be given instructions to conduct the survey in a quiet classroom, and to remind boys that they are free to refrain from answering any questions they do not want to. Boys will be instructed to complete their survey individually and to not look at others’ responses. Boys’ data will be collected using Qualtrics software, and teacher data will be collected using checklists in electronic (a Microsoft Word document) or paper form. Boys will be given a unique code so that their data can be matched over time and remain anonymous, with code lists stored separately from the data. Teachers’ data will only be identifiable to the research team. Upon completion of each stage of data collection, boys will receive information about how to seek support for any distress they may feel, including contact details for free and confidential support services and their school counsellor. Data collection forms can be requested from the research team.

### Data management

All student participant data will be entered electronically using Qualtrics software, and will be stored on password protected Qualtrics accounts accessible only to the researchers. Downloaded data will be stored on a password protected file accessible only to the researchers. All teacher data gathered either in paper format or electronically (a Microsoft Word document) will be transferred to a data sheet by the research team, and stored in a password protected file.

### Data analysis

The effectiveness of the intervention will be primarily determined through conduct of 16 generalized linear three-level mixed-effects regression models predicting our primary and secondary continuous outcome variables and two three-level logistic regression models predicting our primary categorical outcome variables. The effect of primary interest in these models is the fixed-effect of the intervention at the post-intervention and follow-up time points (i.e., the second and third time points, respectively). We will examine and account for potential clustering effects at both the school and classroom level. Missing data will be estimated using full-information maximum likelihood modelling. Within these models, we will additionally conduct mediation analyses to establish whether, and to what extent, the following variables mediate change in our dependent variables: internalisation – muscular, and internalisation – low body fat. Deviations from the above-described analysis plan will be fully described and justified.

To determine the required sample size for these analyses we conducted a comprehensive power analysis using RMASS (Roy, Bhaumik, Aryal, & Gibbons, 2007). We based our analysis on the following parameters; 3 measurement occasions (pre, post, followup), a conservative attrition rate of 5% from pre to post and 15% from post to 2 month follow up (which accounts for the longer time interval from post to followup), a small to medium effect size representing the hypothesized effectiveness of the intervention, equal numbers of students in the control and intervention arms, and a conventional alpha of .05. To achieve a power of .80, the analysis indicated that 504 students (252 in each of the intervention and control) were required. Thus, with a sample size of 600 the study is sufficiently powered with an appropriate amount of participants in case of natural attrition and unanticipated events that reduce participant numbers (e.g., student absence due to illness).

### Data monitoring and harms

Regular checks of data that include items to indicate harm (i.e., a single-item question about distress, as well as an open-ended write-in option) will be regularly monitored by the research team to ensure that the trial is not causing distress to participants. The research team will discuss any responses that indicate distress caused by the study that was not pre-existing (i.e., not pre-existing body dissatisfaction), should this occur, and take appropriate action including either modifying the program or stopping the trial, depending on the response. The team is also required to produce a yearly report to the Victoria University Human Ethics Committee. The Victoria University Human Ethics Committee is independent from all members of the research team.

## Strengths and limitations of the study

Boys have typically been overlooked in body image research, and there are few effective intervention programs for boys. There is evidence that body image, the use of supplements, and attitudes towards doping in sport are interrelated and contribute to anabolic steroid use in adolescent boys. The theoretical frameworks underpinning body image and intentions to use supplements are very different for boys and girls. We will focus only on boys in order to confirm approaches that can be used to target these behaviours in a single sex setting to fill this gap in the literature. This research is novel in that a) it is a boys-only body dissatisfaction prevention program, and b) it is one of the few programs focusing on both body image and APES.

Another strength of this trial is the preliminary studies that have been conducted in order to inform the intervention, both from a theoretical and practical perspective. A great deal of our intervention development has been documented, and this will be beneficial to researchers who wish to develop similar programs of their own.

Although we are using a cluster RCT as opposed to a traditional RCT, the practicality of running a traditional RCT within a classroom setting is poor – as such, a cluster RCT is appropriate and will result in increased generalisability. Teacher delivery of the program is simultaneously a strength and a weakness; while this will improve our generalisability, we cannot be certain that all teachers will deliver the program with the same skill level or adherence to program activities. As such, it was important for us to include teacher evaluation and feedback.

## Issues for ethical review and approvals

The research has been approved by the Victoria University HREC (18–027). Additional approval to conduct the research in Victorian public schools has been granted by the Department of Education and Training, approval number 2018_003920. Model consent forms can be viewed at https://anzctr.org.au/Trial/Registration/TrialReview.aspx?ACTRN=12619000725167

### Protocol description and data availability

Protocol deviations, including sample size, program content, study populations, or other large modifications will be described in any publications resulting from the program. Data will not be available to the public, as participants are under 18 and we wish to maintain their total privacy as well as the privacy of the schools they attend. The final dataset will be accessible only to the research team.

### Access to the program and findings

To ensure that access to the program is equitable, the control group is a waitlist control group and their teachers will facilitate the program after completing follow-up measures. We intend to disseminate the research through a) publications in peer-reviewed journals, b) conference presentations, and c) reports to schools and organisations relevant to HPE in schools (e.g., ACHPER).

### Authorship eligibility guidelines

Topics for manuscripts or conference presentations will be presented to all authors for discussion, and the order of authorship will be discussed. Authorship order will be determined by amount of contribution to the manuscript writing and design. All authors will be named on papers where they have read, contributed to, and approved the manuscript due to the contribution of all research team members (ZY, SM, SG, and JD) at all stages of the research.

## Discussion

As boys’ body image is becoming increasingly recognised as an issue, it is essential that programs are developed specifically with boys in mind, and trialled to examine their efficacy. Additionally, emerging literature on the role of APES, supplements and steroids in body image [34, 51] highlights the importance of prevention and early intervention, and to our knowledge, GoodForm is the first program to address both for an adolescent male audience. We anticipate that the data from GoodForm will provide a valuable addition to the literature on boys’ body image prevention programs, and contribute to an understanding of the development and mechanisms of such programs.

Although there is capacity to discuss Appearance and Performance Enhancing supplements within the Australian Curriculum, very few resources exist to facilitate education about these substances or to facilitate changes in attitudes and behaviours towards APES in the school setting. Traditional resources to support education about performance-enhancing drug use in sport are generally developed along a moralistic, and value-based framework, rather than one that addresses the psychological and behavioural drivers of supplement use. Strengths of the program include alignment with the Australian HPE curriculum for Year 9 and 10, and as such, this program can be incorporated into regular HPE lessons, facilitating broad dissemination of this evidence-based resource.

Following the full Randomised Controlled Trial of this program, we will incorporate teacher feedback to refine and update the program, formalise the teacher education materials, and then engage in broad dissemination of the program through online platforms using best practice implementation science frameworks for mental health programs in schools [52].

## Data Availability

Not applicable.

## Abbreviations

APES: Appearance and performance enhancing substances
HPE: Health and physical education

## References

[1] Goldberg L, Elliot DL, Clarke GN, MacKinnon DP, Zoref L, Moe E (1996). The adolescents training and learning to avoid steroids (ATLAS) prevention program: background and results of a model intervention. Arch Pediatr Adolesc Med.

[2] Stice E, Mazotti L, Weibel D, Agras WS (2000). Dissonance prevention program decreases thin-ideal internalization, body dissatisfaction, dieting, negative affect, and bulimic symptoms: a preliminary experiment. Int J Eat Disord.

[3] Murray SB, Nagata JM, Griffiths S, Calzo JP, Brown TA, Mitchison D (2017). The enigma of male eating disorders: a critical review and synthesis. Clin Psychol Rev.

[4] Fingeret MC, Warren CS, Cepeda-Benito CS, Gleaves DS (2006). Eating disorder prevention research: a Meta analysis. Eat Disord.

[5] Stice E, Shaw H, Marti CN (2007). A meta-analytic review of eating disorder prevention programs: encouraging findings. Annu Rev Clin Psychol.

[6] Wilksch SM, Wade TD (2009). School-based eating disorder prevention.

[7] Yager Z, Diedrichs PC, Ricciardelli LA, Halliwell E (2013). What works in secondary schools? A systematic review of classroom-based body image programs. Body Image.

[8] Brown TA, Forney KJ, Pinner D, Keel PK (2017). A randomized controlled trial of the body project: more than muscles for men with body dissatisfaction. Int J Eat Disord.

[9] Brown TA, Keel PK (2015). A randomized controlled trial of a peer co-led dissonance-based eating disorder prevention program for gay men. Behav Res Ther.

[10] Kilpela LS, Blomquist K, Verzijl C, Wilfred S, Beyl R, Becker CB (2016). The body project 4 all: a pilot randomized controlled trial of a mixed-gender dissonance-based body image program. Int J Eat Disord.

[11] Wilksch SM, Wade TD (2009). Reduction of shape and weight concern in young adolescents: a 30-month controlled evaluation of a media literacy program. J Am Acad Child Adolesc Psychiatry.

[12] Yager Z, O'Dea J (2010). A controlled intervention to promote a healthy body image, reduce eating disorder risk and prevent excessive exercise among trainee health education and physical education teachers. Health Educ Res.

[13] Wilksch SM, Paxton SJ, Byrne SM, Austin SB, McLean SA, Thompson KM (2015). Prevention across the Spectrum: a randomized controlled trial of three programs to reduce risk factors for both eating disorders and obesity. Psychol Med.

[14] Bird E, Halliwell E, Diedrichs PC, Harcourt D (2013). Happy being me in the UK: a controlled evaluation of a body image intervention with pre-adolescent children. Body Image.

[15] McCabe MP, Connaughton C, Tatangelo G, Mellor D, Busija L (2017). Healthy me: a gender-specific program to address body image concerns and risk factors among pre-adolescents. Body Image.

[16] Wilksch Simon M. (2013). School-based eating disorder prevention: a pilot effectiveness trial of teacher-deliveredMedia Smart. Early Intervention in Psychiatry.

[17] Diedrichs PC, Atkinson MJ, Steer RJ, Garbett KM, Rumsey N, Halliwell E (2015). Effectiveness of a brief school-based body image intervention ‘dove confident me: single session’ when delivered by teachers and researchers: results from a cluster randomised controlled trial. Behav Res Ther.

[18] Kater KJ, Rohwer J, Londre K (2002). Evaluation of an upper elementary school program to prevent body image, eating and weight concerns. J Sch Health.

[19] Jankowski GS, Diedrichs PC, Atkinson MJ, Fawkner H, Gough B, Halliwell E (2017). A pilot controlled trial of a cognitive dissonance-based body dissatisfaction intervention with young British men. Body Image.

[20] Stanford JN, McCabe MP (2005). Evaluation of a body image prevention programme for adolescent boys. Eur Eat Disord Rev.

[21] McCabe MP, Ricciardelli LA, Karantzas G (2010). Impact of a healthy body image program among adolescent boys on body image, negative affect, and body change strategies. Body Image.

[22] Levine MP, Smolak L (2006). The prevention of eating problems and eating disorders: theory, research and practice.

[23] Yager Z, Diedrichs PC, Drummond MJN (2013). Understanding the role of gender in body image research settings: participant gender preferences for researchers and co-participants in interviews, focus groups, and interventions. Body Image.

[24] Strother E, Lemberg R, Stanford SC, Turberville D (2012). Eating disorders in men: underdiagnosed, undertreated, and misunderstood. Eat Disord.

[25] Richardson SM, Paxton SJ, Thomson JS (2009). Is BodyThink an efficacious body image and self-esteem program? A controlled evaluation with adolescents. Body Image.

[26] Wilksch SM, Wade TD (2013). Life smart: a pilot study of a school-based program to reduce the risk of both eating disorders and obesity in young adolescent girls and boys. J Pediatr Psychol.

[27] IDEO (2015). The Field Guide to Human-Centred Design.

[28] Kemmis S, McTaggart R (2005). Participatory Action Research: Communicative Action and the Public Sphere. The Sage handbook of qualitative research.

[29] Brown T, Wyatt J (2010). Design thinking for social innovation by. Stanf Soc Innov Rev.

[30] Halprin D (2019). Parent perspectives on improving education for boys about image and performance-enhancing drugs and supplements: An exploratory study. [Masters Thesis].

[31] Walker JR (2019). Action research for Goodform, an interactive intervention program to improve adolescent boys’ body dissatisfaction, attitudes towards doping in sport and intentions to use muscle-building supplements. [Masters Thesis].

[32] Doley JR, McLean SA, Griffiths S, Yager Z. Designing body image and eating disorder prevention programs for boys and men: Theoretical, practical and logistical considerations from boys, parents, teachers, and experts. Manuscript submitted for publication. 2019.

[33] Hazzard Vivienne M, Borton Kelley A, Bauer Katherine W, Sonneville Kendrin R (2017). Cross-sectional associations between gender-linked personality traits and use of weight-loss and muscle-building products among U.S. young adults. Eating Disorders.

[34] Yager Z, O'Dea J (2014). Body image, nutritional supplement use, and attitudes towards doping in sport among adolescent boys: Implications for prevention programs. JISSN.

[35] Goldberg L, Elliot D, Clarke GN, MacKinnon DP, Moe E, Zoref L (1996). Effects of a multidimensional anabolic steroid prevention intervention: the adolescents training and learning to avoid steroids (ATLAS) program. JAMA.

[36] Yager Z, McLean SA, Li X (2019). Body image outcomes in a replication of the ATLAS program in Australia. Psychol Men Masculinity.

[37] Bandura A, Walters RH (1977). Social learning theory: prentice-hall Englewood cliffs, NJ.

[38] Berkowitz AD (2005). An overview of the social norms approach. Changing the culture of college drinking: A socially situated health communication campaign.

[39] Becker CB, Smith LM, Ciao AC (2005). Reducing eating disorder risk factors in sorority members: a randomized trial. Behav Ther.

[40] Mc Laughlin GH (1969). SMOG grading-a new readability formula. J Read.

[41] Ryan TA, Morrison TG, Roddy S, McCutcheon J (2011). Psychometric properties of the revised male body attitudes scale among Irish men. Body Image.

[42] Tylka TL, Bergeron D, Schwartz JP (2005). Development and psychometric evaluation of the male body attitudes scale (MBAS). Body Image.

[43] Griffiths S, Murray SB, Krug I, McLean SA (2018). The contribution of social media to body dissatisfaction, eating disorder symptoms, and anabolic steroid use among sexual minority men. Cyberpsychol Behav Soc Netw.

[44] Kelly NR, Cotter E, Guidinger C (2018). Men who engage in both subjective and objective binge eating have the highest psychological and medical comorbidities. Eat Behav.

[45] Schaefer LM, Harriger JA, Heinberg LJ, Soderberg T, Kevin TJ (2017). Development and validation of the sociocultural attitudes towards appearance questionnaire-4-revised (SATAQ-4R). Int J Eat Disord.

[46] Matera C, Nerini A, Stefanile C (2019). Sexual orientation, peer influence, body dissatisfaction, and Eudaimonic well-being in Italian men. Front Psychol.

[47] Parent MC, Moradi B (2011). An abbreviated tool for assessing conformity to masculine norms: psychometric properties of the conformity to masculine norms Inventory-46. Psychol Men Masculinity.

[48] Ling J, Robbins LB, Resnicow K, Bakhoya M (2014). Social support and peer norms scales for physical activity in adolescents. Am J Health Behav.

[49] Sladek M, Engeln R, Miller S (2014). Development and validation of the male body talk scale: a psychometric investigation. Body Image.

[50] McLean SA, Wertheim EH, Marques MD, Paxton SJ (2019). Dismantling prevention: comparison of outcomes following media literacy and appearance comparison modules in a randomised controlled trial. J Health Psychol.

[51] Hazzard VM, Borton KA, Bauer KW, Sonneville KR (2018). Cross-sectional associations between gender-linked personality traits and use of weight-loss and muscle-building products among US young adults. Eat Disord.

[52] Lyon AR, Bruns EJ (2019). User-centered redesign of evidence-based psychosocial interventions to enhance implementation-hospitable soil or better seeds?. JAMA Psychiatry.

